# Antibody Subclass Repertoire and Graft Outcome Following Solid Organ Transplantation

**DOI:** 10.3389/fimmu.2016.00433

**Published:** 2016-10-24

**Authors:** Nicole M. Valenzuela, Michelle J. Hickey, Elaine F. Reed

**Affiliations:** ^1^UCLA Immunogenetics Center, University of California Los Angeles, Los Angeles, CA, USA; ^2^Department of Pathology and Laboratory Medicine, David Geffen School of Medicine, University of California Los Angeles, Los Angeles, CA, USA

**Keywords:** IgG subclass, HLA donor-specific antibodies, transplant

## Abstract

Long-term outcomes in solid organ transplantation are constrained by the development of donor-specific alloantibodies (DSA) against human leukocyte antigen (HLA) and other targets, which elicit antibody-mediated rejection (ABMR). However, antibody-mediated graft injury represents a broad continuum, from extensive complement activation and tissue damage compromising the function of the transplanted organ, to histological manifestations of endothelial cell injury and mononuclear cell infiltration but without concurrent allograft dysfunction. In addition, while transplant recipients with DSA as a whole fare worse than those without, a substantial minority of patients with DSA do not experience poorer graft outcome. Taken together, these observations suggest that not all DSA are equally pathogenic. Antibody effector functions are controlled by a number of factors, including antibody concentration, antigen availability, and antibody isotype/subclass. Antibody isotype is specified by many integrated signals, including the antigen itself as well as from antigen-presenting cells or helper T cells. To date, a number of studies have described the repertoire of IgG subclasses directed against HLA in pretransplant patients and evaluated the clinical impact of different DSA IgG subclasses on allograft outcome. This review will summarize what is known about the repertoire of antibodies to HLA and non-HLA targets in transplantation, focusing on the distribution of IgG subclasses, as well as the general biology, etiology, and mechanisms of injury of different humoral factors.

## Introduction

The clinical significance of antibody-mediated rejection (ABMR) to transplant outcome is now widely established across solid organ transplants. The recent introduction of solid-phase single antigen antibody testing by Luminex enabled detection of donor-specific antibodies with significantly greater sensitivity than had been previously available. Advances in T cell immunosuppression have significantly reduced T cell-mediated rejection as a cause of graft loss in medication adherent patients; consequently, antibody-mediated rejection has emerged as the leading cause of allograft failure (1).

Transplant recipients may be transplanted with either preformed donor-specific antibodies (DSA) that were generated by prior allosensitization events, such as pregnancy, transfusion, or transplantation, or develop DSA *de novo* after transplantation. Wiebe and colleagues reported (2) that low-risk renal transplant recipients develop *de novo* DSA at a rate of about 2% per year, appearing usually around 2 years post-transplant. By 12 years post-transplant, the final incidence of DSA was 27%. Similar rates of *de novo* DSA were reported by Everly et al., wherein 25% of patients had DSA by 10 years post-transplant (3). Pediatric and adult heart transplant recipients developed *de novo* DSA with an incidence of about 30–40% by 10 years post-transplant (4–6). Liver (7–9), lung (10, 11), pancreas (12, 13), and bowel (14, 15) transplant recipients also develop donor-specific human leukocyte antigen (HLA) antibodies.

Overall, DSA are seen in ~20% of solid organ transplant recipients and are a significant clinical factor in transplant outcomes. Diagnostic criteria for ABMR vary slightly across solid organs, although endothelial cell injury, complement deposition, and mononuclear cell infiltration are recurrent manifestations. In renal transplants, acute ABMR is defined by histological evidence of tissue injury, such as microvascular inflammation (MVI) or arteritis, with or without complement C4d staining, and serological evidence of DSA (16, 17). Chronic rejection of renal allografts may also be triggered by donor-specific antibodies and is characterized by transplant glomerulopathy, capillary basement membrane duplication or fibrosis, and MVI (17). In cardiac allografts, histologic changes, including endothelial cell activation and intravascular CD68^+^ macrophages, as well as complement activation detected by C4d or C3d deposition, are included in the diagnosis of pathologic ABMR (18, 19). ABMR in lung (20), pancreas (21), and liver (22) allografts also include a combination of C4d staining, mononuclear cell infiltration, and histological assessment of microvessel endothelial cell activation.

Transplant recipients developing DSA to polymorphic HLAs exhibit significantly worse graft survival and rejection rates. Allograft loss was higher in renal transplant patients who developed *de novo* DSA compared with patients who did not and had no dysfunction, and interestingly, patients could be further stratified by concurrent clinical ABMR at the time of DSA appearance. Those with subclinical DSA fared worse in the long-term than those without any DSA, but significantly better than those who had clinical ABMR at the detection of DSA, all of who lost their allografts by 8 years after the appearance of DSA. While non-adherence and delayed graft function (DGF) were significant predictors of graft loss, the strength or MFI of DSA was not itself a strong predictor (2, 23). Pediatric heart transplant recipients with DSA have higher incidences of cardiac allograft vasculopathy (CAV), also called transplant coronary artery disease (TCAD), compared to those without DSA, more rejection episodes, and lower graft survival at 5 years (5, 6). In adult heart transplant recipients, DSA is also an independent predictor of patient survival (4). Many studies have also demonstrated a clear decrement in outcome and graft survival among patients with antibody to non-HLA targets, such as major histocompatibility complex class I chain-related gene A (MICA) (24–27).

In spite of overwhelming evidence that patients with DSA tend to fare worse as a group than those without, these same studies have consistently shown that up to half (range 20–50%) of patients with HLA DSA do *not* experience poorer graft outcomes, including rejection incidence and graft loss, compared to their DSA^−^ counterparts, at least to the endpoints reported (5, 6, 28–30). Indeed, even among DSA^+^ patients with adverse outcomes, there is a spectrum from subclinical, “indolent” antibody-mediated graft injury to clinically manifested acute antibody-mediated rejection to devastating hyperacute rejection. This has led to the hypothesis that not all antibodies are equally pathogenic and that identification of antibody features controlling graft injury might enable further stratification of patients with DSA who are at risk for rejection and allograft failure. Certainly, the titer of antibody is important for the degree of graft injury. The affinity of an antibody for its antigen and the effector functions an antibody can engage are also likely to be relevant to its “pathogenicity” in antibody-mediated allograft injury. Such diversity in antibody function is controlled in large part by the antibody’s isotype and subclass. This review will discuss the function of different IgG subclasses relevant to graft injury, the subclass repertoire of HLA and non-HLA antibodies found in transplantation, and the generation of antibodies of various isotypes and subclasses.

## Diversity of Effector Functions among IgG Subclasses

The heavy chain constant regions of human IgG are more than 90% homologous, with variation mostly localizing to the CH2 domain and hinge regions. Not coincidentally, these are the locations for Fc-mediated effector engagement, chiefly binding to FcγRs and complement C1q. Immunoglobulin effector functions are engaged by the Fc portion of IgG and include complement-dependent cytotoxicity (CDC), opsonization, antibody-dependent cell-mediated cytotoxicity (ADCC), and antibody-dependent cell-mediated phagocytosis. IgG subclasses vary in the length and flexibility of their hinge region, with IgG2 and IgG3 representing opposite ends of the spectrum. The hinge region is the shortest and most rigid in IgG2, while IgG3 has a uniquely long hinge region that is quite flexible, promoting increased availability to the binding sites for FcγR and C1q. Studies optimizing effector functions of therapeutic antibodies have also pinpointed key amino acid residues, which control affinity of the Fc region for FcγRs and for complement components.

IgG4 has been called an “odd antibody” due to its unique structural properties (31). IgG4 can form Fab arm monomers (half-molecules) that are monovalent. This feature is thought to represent a self-limiting process of the humoral immune response, as these monomers can block better effector subclasses from binding to antigen. Another consequence of reduced stability in the IgG4 Fc tail is its reported ability to exchange Fab arms and form bispecific antibody molecules.

### Affinity, Persistence, and Localization

Some evidence points to differences in IgG subclass affinity for antigens. Berkowska et al. found that in peripheral blood B cells, transcripts of IGHG2 and IGHG4 contained higher loads of somatic hypermutation in the variable regions compared with IGHG1 and IGHG3 (32). Thus, “indirect” sequential switching to IgG2 and IgG4 might indicate more germinal center reactions and longer activity of the enzyme activation-induced cytidine deaminase (AID). It might be surmised from this work that later subclasses IgG2 and IgG4 result from more extensive affinity maturation compared with upstream IgG1 and IgG3 (32), as was suggested by early work evaluating IgG subclass response and affinity in human KLH immunization (33). These results are similar to findings that mutation loads were highest in antigen-specific IgG4 compared with other subclasses after secondary immunization with *Meningococcus* (34). Studies of murine IgG subclasses reveal similar differences in affinity. One study put forth the hypothesis that the constant region itself affected affinity for antigen, where IgG of different subclasses, but with identical variable regions, displayed different affinity for antigen (35–37). Antibody affinity (i.e., variable region variants) for antigen appears to be important for its ability to provoke Fc-mediated effector functions such as ADCC, discussed below (38, 39).

IgG3 has the shortest half-life, of about a week, while the other subclasses have a half-life of about 3 weeks. This is due to an arginine at amino acid position 435 of IgG3, rather than a histidine which is present in other subclasses, significantly increasing its affinity for the neonatal Fc salvage receptor (FcRn) (40). Interestingly, although many allotypes of immunoglobulin have been identified to date (41), only polymorphisms in IgG3 which revert this position to histidine demonstrate any change in function, increasing the half-life of these alleles of IgG3 (42). The affinity of IgG subclasses for FcRn is of relevance to antibody-mediated diseases of the fetus and newborn. In addition to variation in placental transport, the isotypes of antibodies also differ in their ability to diffuse into the host’s own tissues, which serves to compartmentalize the humoral response. IgM has poor diffusion due to its size, whereas IgA is found in secretions and at epithelial surface. IgG is predominantly in circulation but also can diffuse into the tissues. Traditionally, IgG3 was described to have poor transport across the placenta due to its low affinity for FcRn; however, it was recently emphasized that the majority of IgG3 alleles represented in Western study populations were R435, in contrast to the H435 found frequently in other groups. Therefore, ethnic differences in IgG allotypes are also important to antibody effector functions.

### Agonism

**Figure 1:**
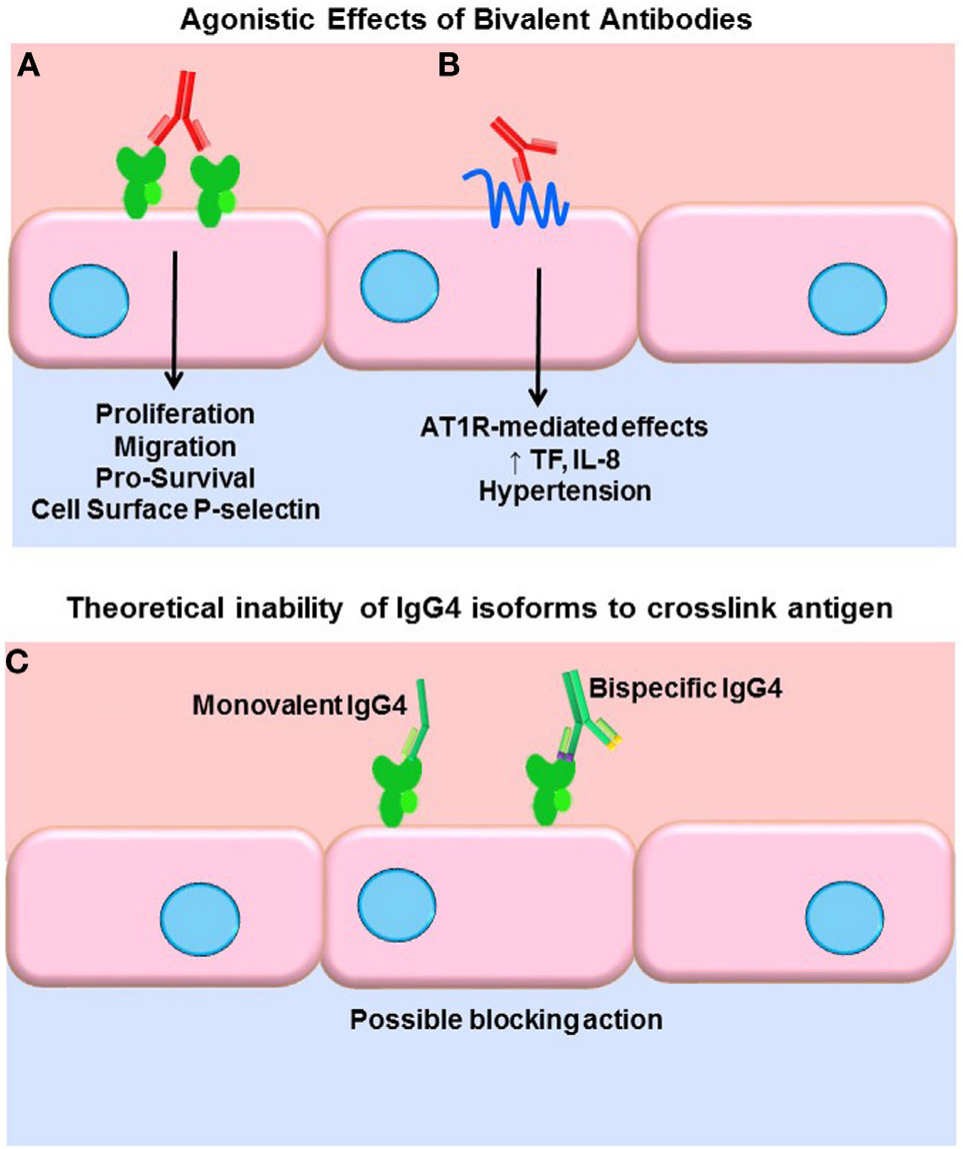
**Agonistic signaling by antibodies**. **(A)** Bivalent IgG of any subclass may dimerize or crosslink target antigens, such as HLA molecules. Many studies have demonstrated that HLA ligation on endothelial cells, vascular smooth muscle cells, and antigen-presenting cells induces intracellular signaling *via* tyrosine kinases. HLA signaling promotes cell proliferation, migration, and increased expression of survival proteins such as Bcl-2 and Bcl-XL. In addition, HLA cross-linking triggers exocytosis of endothelial vesicles called Weibel–Palade bodies, which contain vasoactive mediators and the adhesion molecule P-selectin. Increased cell surface P-selectin in turn supports increased adherence of leukocytes. **(B)** Anti-AT1R antibodies act agonistically, binding to an epitope on this multi-pass transmembrane receptor and stimulating increased IL-8 expression and tissue factor production. AT1R agonism is also implicated in malignant hypertension in a variety of diseases as well as transplantation. **(C)** Some molecules of human IgG4 have been shown to form monovalent Fab arms that may cross-dimerize with other clones of IgG4 to create bispecific antibodies. Although not experimentally demonstrated, in theory, such monovalent and bispecific IgG4 molecules would be incapable of cross-linking HLA and might in fact block other subclasses from binding.

A key function of antibodies is to block their target, neutralizing it as with viruses. IgG, being bivalent, can also dimerize its target antigen and stimulate inhibitory or agonistic signaling depending on the target. Our group and others have shown that antibodies to HLA class I molecules also activate intracellular signaling cascades in vascular endothelial and smooth muscle cells, resulting in increased cell proliferation, migration, and recruitment of leukocytes (43–54) (Figure 1A). Less is known about agonistic signaling of antibodies against HLA II molecules. In antigen-presenting cells, HLA-DR ligation by antibodies mimics TCR engagement, and induces cell activation and proliferation (55–59). Endothelial cells expressing HLA-DR also respond to anti-HLA-DR antibodies by increased allostimulation of T cells (60, 61). Transcriptome studies of renal transplant biopsies undergoing ABMR have revealed an enriched endothelial-specific signature, paralleling these *in vitro* studies (62). Antibodies to the angiotensin II type 1 receptor (AT1R) agonistically activate AT1R signaling and induce detrimental effects on vascular endothelial phenotype and function (63, 64) (Figure 1B). AT1R antibodies are implicated in systemic sclerosis (SSc), preeclampsia (65), hypertension (66), and allograft dysfunction (67, 68). Auto-antibodies to endothelial cells [anti-endothelial cell antibodies (AECA)], against yet mostly unidentified antigens, activate endothelia to express a variety of adhesion molecules (69).

Functional target agonist activity is presumably independent of the Fc portion of the antibody, as stimulation of endothelium with the F(ab′)_2_ fragment still elicits these functional changes. Interestingly, Stein et al. demonstrated that a chimeric anti-HLA-DR hIgG4 (engineered to stay bivalent, not form half-molecules), while not able to induce Fc-mediated functions such as complement activation or ADCC of tumor B cells, significantly increased intracellular Akt signaling, suppressed proliferation, and induced apoptosis at a comparable level as the parental anti-HLA-DR (70). So, while generally incapable of eliciting Fc-mediated effector functions, if bivalent, IgG4 can still cross-link HLA molecules and provoke intracellular signaling cascades and target cell phenotype changes. Therefore, it could be surmised that signaling of HLA and non-HLA targets in the allograft can be induced by all subclasses of immunoglobulin, with the possible exception of monovalent or bispecific forms of IgG4 (Figure 1C). The differential ability of IgG subclasses to induce agonistic signaling remains to be explored.

### Complement-Dependent Cytotoxicity and Inflammation

Arguably, the most studied effector function of antibodies in transplantation is the ability to activate complement. Histological detection of the complement split product C4d within allograft vasculature has been central to the diagnostic criteria of ABMR for decades.

**Figure 2:**
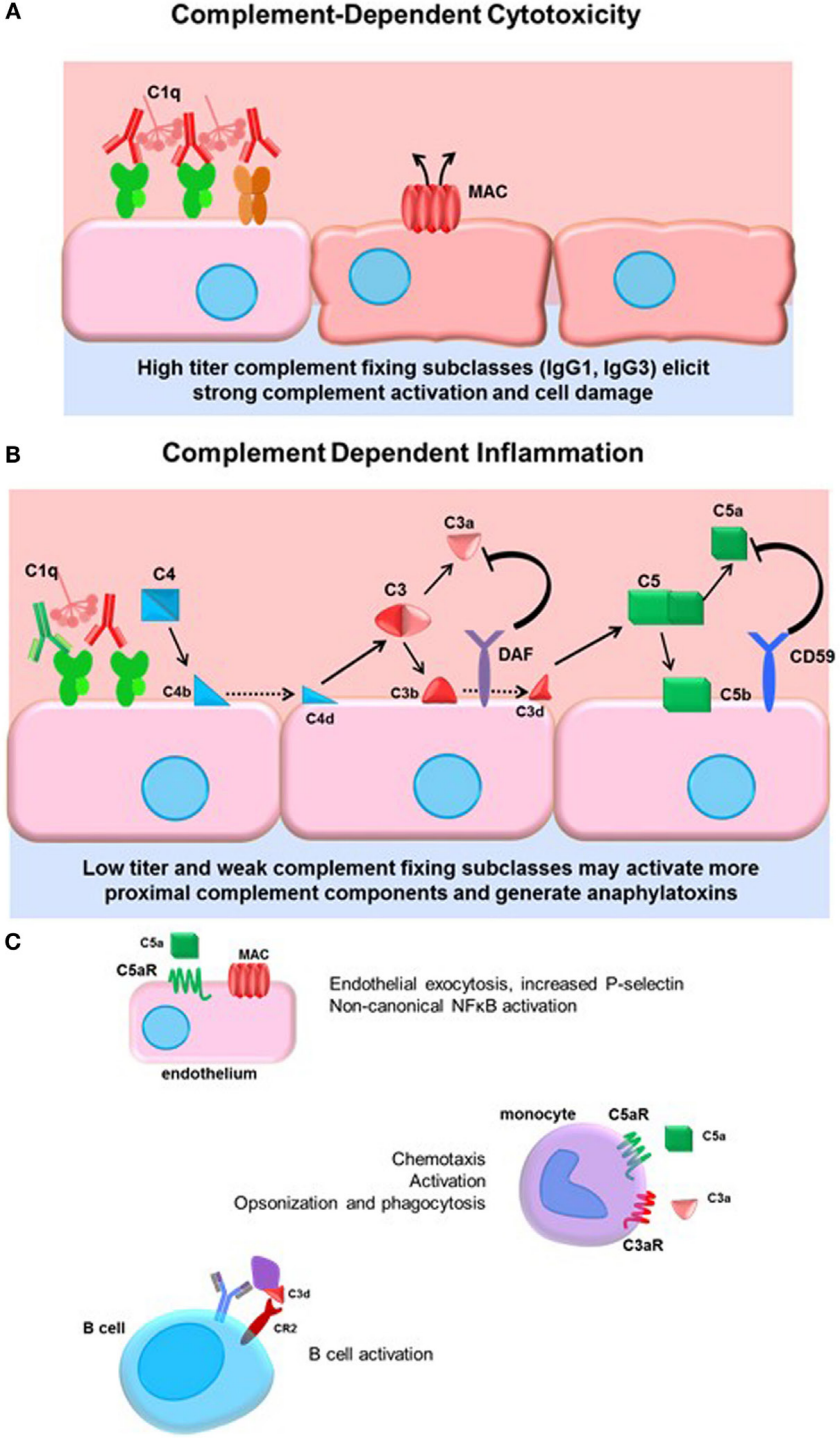
**Complement-dependent cytotoxicity and inflammation**. **(A)** High titers of antidonor HLA antibodies, particularly of the efficient complement-fixing subclasses IgG1 and IgG3, may promote terminal classical complement pathway activation. Complement activation must overcome the regulatory factors and push complement activation to terminal MAC formation and cell damage. **(B)** Lower titers of antibody or less efficient complement-fixing subclasses, such as IgG2, may result in truncated complement activation, with upstream anaphylatoxin release and opsonin deposition. The initiator C1 complex, composed of globular C1q, embedded with catalytically active C1r and C1s, recognizes the Fc portion of IgM and most of the IgG subclasses, triggering a conformational change in the hexameric shape of the C1 complex. This activates the autocatalytic cleavage of C1r, which then activates C1s. C4 and C2 are cleaved by C1s, forming C4a and C2a split products that generate the C3 convertase. C3 convertase cleaves C3 protein into C3a, a soluble inflammatory protein, and C3b, which is covalently bound to the cell surface. C3b may be further cleaved to C3d or form the C5 convertase. Terminal activation of the C5 convertase cleaves C5 protein, generating the potent anaphylatoxin C5a and the membrane-bound C5b. C5b recruits C6–9 proteins to form the membrane attack complex (MAC), disrupting membrane integrity. Complement regulatory proteins DAF and CD59 at the host cell surface restrain activation of the complement cascade at the two key amplification steps, C3 cleavage and C5 cleavage. **(C)** Many cells express receptors for the soluble and membrane-bound complement split products. Endothelial cells respond to C5a by upregulating P-selectin, and to sublytic concentrations of MAC by activation of non-canonical NFκB pathways, adhesion molecule, and cytokine upregulation. Monocytes and neutrophils express C3a and C5a receptors, which participate in chemotaxis of myeloid cells. Complement receptor 2 (CR2) is a component of the BCR that binds to opsonized, C3d-coated antigen. CR2 signaling enhances the BCR signal and lowers the threshold for B cell activation.

The complement system is ancient part of the innate immune system culminating in activation of terminal split products that are highly inflammatory and can cause target cell death (whether mammalian or pathogen). Three arms of the complement system, lectin, alternative, and classical pathways are triggered by different stimuli, but all converge on the central regulator C3. Canonically, the classical pathway is activated by immunoglobulin. The details of the classical complement pathway have been excellently reviewed elsewhere (71); a brief summary can be found in Figure 2.

Activation of complement is balanced by complement regulatory proteins, both soluble and cell surface, and self-limiting due to cleavage of active mediators. Such regulatory proteins, including decay-accelerating factor (DAF, CD55), MCP (CD46), and CD59, expressed on endothelial and other cells, and serum C1-INH and Factor H, limit inflammation and confer protection of host cells during complement activation. DAF acts to inhibit C3 activation downstream of C4 and C2 and upstream of C3 and C5. Anti-EGFR IgG3 promoted deposition of C1q and C3b on target tumor cells, as well as generation of upstream C4a, but failed to result in the generation of C3a or C5a due to high expression of CD55 on the tumor cells (72), demonstrating the resistance of cells to terminal complement activation when they express complement regulatory proteins. Indeed, deficiency of the complement regulatory protein DAF abolishes protection of corneal allografts from alloimmune destruction (73) and exacerbates rejection of cardiac allografts in mice (74, 75). Interestingly, among human cardiac transplant recipients with active C4d^+^ antibody-mediated rejection, those without concurrent dysfunction exhibited increased expression of DAF compared to patients who had ABMR and allograft dysfunction (76). These studies underscore the protective role of complement regulatory proteins in transplantation.

The four subclasses of IgG vary in their affinity for C1q (Table 1). IgG3 is a potent stimulator of complement activation, with IgG1 following closely behind. IgG2, although typically cited as non-complement fixing, in fact can fairly efficiently bind to C1 and activate complement under conditions of high antigen density and/or high antibody titer. IgG4, in contrast, has nearly no detectable complement-activating properties. Experiments using subclass switch variants carrying the same variable regions have shown that IgG4 is a poor activator of human CDC compared with IgG1 and IgG3, with IgG2 having intermediate complement activity at higher antibody concentration (70, 77). Interesting murine models of ABMR showed that non-complement-fixing subclasses of antidonor IgG were able to augment complement activation by the stronger complement-fixing subclasses (78).

**Table 1:** **Summary of IgG subclass effector functions**.

				Binding to FcγR alleles							
	ADCC (NK)	Complement activation	Antigen recognized	FcγRI CD64	FcγRII CD32a-H	FcγRII CD32A-R	FcγRII CD32b	FcγRIII CD16a-V	FcγRIII CD16a-F	FcγRIII CD16b-NA1	FcγRIII CD16b-NA2
IgG1	+++	+++	T-dep protein	+++	++	+++	+	+++	++	+++	+++
IgG2	+	+^b^	Carbohydrate and T-dep protein	−	++	+	−	+	−	−	−
IgG3	+++	+++	T-dep protein	+++	++	+++	++	+++	+++	+++	+++
IgG4	^a^	−	T-dep protein	+++	+	+	±	++	−	−	−

*^a^ADCC elicited by IgG4 depends on the glycosylation pattern of the Fc region*.

*^b^Complement activation by IgG2 depends on the titer of antibody and the density of antigen*.

It is worthwhile to note that the CDC cross-match assay used in transplantation employs rabbit complement rather than human complement. Due to heterophilic antibodies and inter-species interactions between human IgG subclasses and rabbit complement proteins, the CDC is not necessarily reflective of true complement-activating capacity of human antibodies. Indeed, human IgG2, while not a good activator of the classical human complement cascade, effectively triggers activity of rabbit complement (79). Nevertheless, the cytotoxic cross-match generally does reveal antibodies with very high titer and correlates well with clinical outcomes (80).

Terminal complement activation ultimately triggers cell death, and this outcome has been a focus early in transplantation due to its dramatic and devastating injury to the allograft (81) (Figure 2A). Given the protection of the graft endothelium by constitutive expression of complement regulatory proteins, it is conceivable that only very high titers of strongly complement-fixing antibodies can overcome inhibition to cause endothelial cytolysis. Hyperacute rejection is now a rare event due to improved sensitivity of antibody and cross-match tests and general avoidance of strong donor-specific antibodies. Consequently, there has been increased interest in the upstream mediators of the complement system, and the role of “complement-dependent inflammation.” Anaphylatoxins and opsonins C4a, C3a, C3b, and C5a are all critically important in regulating innate inflammation, as well as modulation of adaptive immunity. C4a, C3a, and C5a are chemoattractant for neutrophils and monocytes (82, 83) (Figure 2B). Endothelial cells respond to C5a by releasing intracellular vesicles containing adhesion molecules and vasoactive mediators (45, 84), and to sublytic deposition of membrane attack complex (MAC) by activation of non-canonical NFκB and upregulation of inflammatory genes including VCAM-1 and E-selectin (85, 86) (Figure 2C). Complement may also be implicated in transplant vasculopathy, although arteriopathy also develops in C3-deficient mice (87, 88).

The regulation of adaptive immunity by complement, particularly complement C3 components, has been revealed by several studies. When B cells encounter opsonized antigen coated with C3d, coligation of the BCR with complement receptors CD21 and CD35 lowers the threshold for B cell activation and enhances humoral immunity [reviewed in Ref. (89)]. Heeger and colleagues have expanded our understanding of how complement modulates T cell responses [reviewed in Ref. (90, 91)]. For example, C3a and C5a enhance T cell proliferation and activation, as well as antigen-presenting cell activation.

### It Is Unclear Whether *In Vitro* Complement Fixation Is a Reliable Predictor of Rejection or Graft Loss

Investigators have also utilized modifications of the HLA solid-phase assays to infer the ability of antibodies to activate complement, measuring binding of complement components C1q (30, 92–95), C4d (96–100), or C3d (96, 101, 102) to single antigen beads and to cells. However, reports conflict as to whether such assays provide better resolution of antibody pathogenicity and indeed ability to initiate the complement cascade in these *in vitro* assays seems to still be tied to antibody titer. Certainly, the strength or titer of antibody seems to be linked with its pathogenicity. For example, as recognized early in solid organ transplantation, very high titers of HLA DSA (that can cause a positive CDC cross-match) can trigger hyperacute rejection and early graft dysfunction. Increasing strength of DSA is associated with lower graft function in renal transplant recipients (103). Zeevi et al. found that high titers of HLA DSA were able to fix C1q and were associated with early ABMR in heart transplant patients (104). In contrast, Smith et al. found that both non-complement-fixing and complement-fixing DSA (measured by C4d deposition on single antigen beads) were associated with reduced heart transplant patient survival (4) and did not conclude that complement activation *in vitro* was a useful predictor of more pathogenic DSA. In studies of kidney transplantation, Crespo et al. reported that C1q-fixing DSA had higher MFIs but that C1q positivity did not correlate with outcome in renal transplant recipients, whereas other groups have uncovered an added predictive value of C1q-positive DSA in renal allograft survival (30, 94, 105–107). In conclusion, there is no clear consensus on whether donor-specific antibodies which fix complement in *in vitro* assays better discriminate those that are detrimental to allograft survival; it does appear, though, that antibodies which do not bind to complement in these assays are still relevant to graft outcome.

### FcγR-Mediated Functions

Antibodies can engage Fc receptors present on most hematopoietic cells. FcγRs are highly selective in their affinity for IgG subclasses (Table 1) [reviewed in Ref. (108, 109)]. The receptors for IgG, the FcγR family, are expressed on myeloid and NK cells as well as B cells. All myeloid cells express activating FcγRs, and some coexpress an inhibitory FcγR. There are three major classes of human FcγRs, FcγRI (CD64), FcγRII (CD32), and FcγRIII (CD16), which are differentially distributed on innate and adaptive effector cells. FcγRI is an activating receptor with high affinity for both monomeric and complexed IgG and is present on the major population of monocytes, as well as macrophages and activated neutrophils. FcγRII has two subtypes, FcγRIIa, expressed on monocytes, macrophages, and highly on neutrophils, and the inhibitory FcγRIIb. FcγRII has comparably lower affinity than FcγRI for monomeric IgG but binds efficiently to high avidity ligands of complexed or immobilized IgG. FcγRIIIa is expressed on NK cells and a minor population of monocytes, while FcγRIIIb is expressed on neutrophils.

Polymorphisms in the FcγR system shape individual susceptibility to infectious disease, autoimmunity, and response to antibody-based therapeutics (110–115). While FcγRI has no known polymorphism, FcγRIIa, FcγRIIIa, and FcγRIIIb are each dimorphic, with two alleles having different affinities for IgG (116). For example, the FcγRIIa alleles H131 and R131 differ in their affinity for IgG1, with H131 having higher affinity and the ability to bind to IgG2. Taken together, it is probable that the transplant recipient’s own constellation of FcγR alleles offset or augment the effects of the subclass repertoire during FcγR-mediated injury.

### Opsonization and Phagocytosis

Antibodies act in concert with complement activation to mark target cells and microbes for uptake by phagocytes, a process called opsonization. FcγRs work with complement receptors to elicit phagocytosis. IgG3 most potently induced opsonization of meningococci (chimeric antibodies + human complement) and respiratory burst in PMN induced, with IgG1 slightly less potent and very activity little with IgG2 or IgG4 (77). Therefore, IgG3 and IgG1 are typically thought of as the most potent opsonizing antibodies.

### Antibody-Dependent Cell-Mediated Cytotoxicity

**Figure 3:**
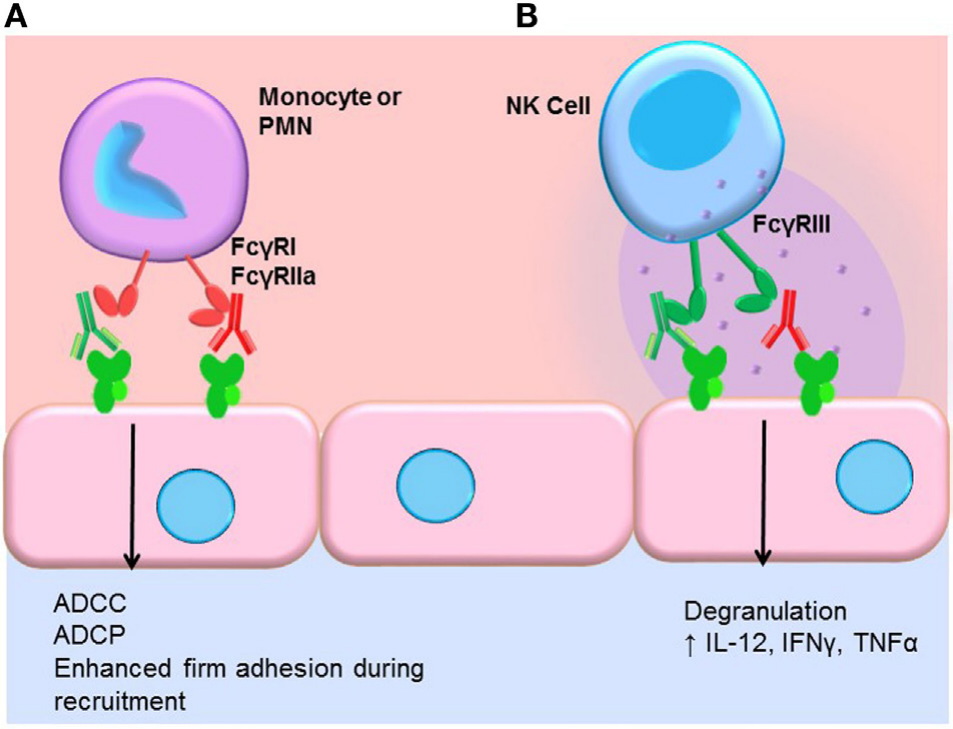
**FcγR-mediated effector functions**. **(A)** Monocytes and neutrophils express FcγRs, which bind preferentially to different IgG subclasses. FcγR cross-linking by myeloid cells induces activation and mediates antibody-dependent cell-mediated cytotoxicity (ADCC), phagocytosis (ADCP), and augments recruitment of leukocytes from the blood. In general, IgG1 and IgG3 efficiently bind to most FcγR isoforms expressed by monocytes and neutrophils. IgG4 can bind quite well to FcγRI (CD64), and IgG2 is bound by an allelic variant of FcγRIIa (CD32a, H131). **(B)** NK cells express FcγRIII (CD16). Engagement of FcγRIII by antibody-coated target cells induces ADCC *via* degranulation and release of perforin/granzyme. Cross-linking of FcγRIII on NK cells also upregulates the inflammatory cytokines IL-12, IFNγ, and TNFα.

Engagement of FcγRs activates ADCC (Figure 3). NK cells express CD16A and CD16C. In NK cells, FcγR cross-linking initiates intracellular signaling leading to polarized release of perforin and granzyme, causing death of the antibody-coated target cell. While myeloid cells, such as macrophages, also carry out ADCC, the mechanisms are less clear. Typically, IgG1 and IgG3 are the most efficient activators of NK cell-mediated ADCC due to the higher affinity of FcγRIIIa for these subclasses (Table 1). Not only is IgG4 ineffective at eliciting ADCC but also has been shown to actively block monocyte-mediated antitumor ADCC when present in equal concentrations with IgG1, through competitive binding to FcγRI (117).

There is limited evidence directly demonstrating that ADCC is actually occurring within allografts during antibody-mediated rejection. How ADCC might manifest histologically is unclear. Early work attempted to prove that alloantibodies were able to induce ADCC against allogeneic endothelial cells, using *in vitro* assays. The authors showed that sera of only a few post-transplant patients were able to induce lysis of cultured endothelial cells by NK cells (118). A follow-up study suggested that patients whose serum was capable of inducing ADCC against cultured endothelial cells had more vascular rejection and graft loss than patients without ADCC activity (119). Experimental models have shown that NK cells are critical for chronic antibody-mediated rejection in the mouse [reviewed in Ref. (120, 121)]. Hidalgo et al. identified NK transcript signatures in renal transplant biopsies from patients with rejection, particularly late antibody-mediated rejection (122, 123). Taken together, these results point to a role for NK cells in antibody-mediated rejection beyond ADCC.

### FcγR Signaling

We and others have shown that FcγR signaling in monocytes and neutrophils participates in the leukocyte recruitment cascade (124, 125). Concurrent engagement of FcγRs and adhesion molecules augments firm adhesion of myeloid cells through increased activation of integrins, enabling increased capture of FcγR-bearing leukocytes by antibody-coated endothelium (Figure 3). FcγR signaling also influences macrophage differentiation, dendritic cell maturation, and enables prolonged antigen presentation. Thus, subclasses that more effectively engage FcγRs on macrophages, neutrophils, and dendritic cells should be better capable of inducing FcγR signaling in these cells.

## Antibody Subclasses in Transplantation

Different routes of allosensitization trigger distinct patterns of IgG subclasses directed against HLA, which supports the paradigm that the inflammatory milieu upon antigen exposure, as well as the antigen itself, controls selection of subclass. Intriguingly, memory formation also appears to differ after various allosensitization events, pointing to immunologically distinct mechanisms of immunization against HLA and MICA through transfusion, pregnancy, and transplantation.

### Assays to Identify HLA Antibodies

Cell-based and solid-phase testing for HLA antibodies classically identify antibodies of the IgG isotype as donor-specific IgG HLA antibodies. The classical complement-dependent cytotoxic assay identifies strong anti-HLA IgG antibodies that bind HLA on the surface of the target cell and initiate the complement cascade culminating in the formation of the MAC complex and cell death identified by fluorescent microscopy. The T and B flow cross-match identifies anti-HLA IgG binding to the surface of T or B cells and quantitates the median channel shift over cells incubated with a negative control serum. In comparison to the CDC, the T and B flow cross-match is more sensitive and quantitative allowing for the identification of antibodies that are weak/moderate in strength. Solid-phase assays identify HLA antibody that bind HLA antigen bound to a plate (ELISA) or bead (single antigen bead) in a cell-free environment. The single antigen bead assay currently allows the most quantitative and sensitive measurement of HLA IgG antibodies identified by Luminex technology.

### Antibody Subclasses in Allosensitization (Pretransplant)

Significant effort has been contributed to determining the IgG subclass repertoire in pre- and post-transplant patients (Table 2). To achieve these goals, laboratories have developed protocols that modify the traditional single antigen bead assay by replacing the IgG detection antibody with subclass-specific clones for IgG1–4 (126–130). The data are consistent in showing IgG1 as the predominant Ig subclass in pre- and post-transplant sera, and have attempted to associate specific subclasses with sensitizing events, allograft pathology, and loss (see below) (126–130).

**Table 2:** **Summary of different methods for detecting IgG subclasses against HLA and non-HLA targets**.

Reference	Organ/target	Determinants of positive cutoff	Antibody source
Lefaucheur et al. (130)	Renal/HLA	For each IgG subclass antibody and each single antigen bead, the mean MFI values from 4 non-sensitized healthy male controls +5 SD, and if that value was <500 MFI; the IgG 1–4 reactivity was considered positive if the normalized MFI was ≥500	Southern Biotech
Khovanova et al. (129)	Renal/HLA	>5× the greater than the average negative control bead MFI in the single antigen bead assay of all tests for each subclass. IgG1: 120.6, IgG2: 72.0, IgG3: 62.7, IgG4:17.2	Southern Biotech
Lowe et al. (128)	Renal/HLA	>5× the greater than the average negative control bead MFI in the single antigen bead assay of all tests for each subclass. IgG1: 120.6, IgG2: 72.0, IgG3: 62.7, IgG4:17.2	Southern Biotech
Kaneku et al. (127)	Liver/HLA	Normalized trimmed MFI higher than 500 was defined as positive on the basis of binding patterns after validation and dilution experiments	Southern Biotech
Honger (126)	Renal/HLA	A positive result was defined by a MFI value above a cutoff that was generated for each IgG subclass and for every individual bead by using four negative control sera from healthy non-sensitized and HLA antibody-negative men: cutoff MFI = mean NC1–4 + 3 SDs NC1–4. To determine the amount of IgG subclasses, we used the ratio above the corresponding cutoff (i.e., ratio = MFI IgG subclass divided by MFI cutoff)	Southern Biotech
Jackson et al. (131)	Endothelial cells	Median fluorescence values for IgG subclasses identified in test serum were normalized to values obtained when cells were incubated with normal control AB serum (Atlanta Biologicals, Norcross, GA, USA). IgG subclass analysis of HLA antibodies was assessed in the same manner using 10 ECP donors and pooled sera from high calculated panel reactive antibody (cPRA) transplant candidates	Southern Biotech
Griffiths et al. (132)	Renal/HLA	For each IgG subclass and for every individual bead by using four negative control sera (NC1–4) from healthy, non-sensitized, and HLA antibody-negative cutoff MFI mean NC1–4 + 3 SDs NC1–4. To determine the amount of IgG subclasses, we used the ratio above the corresponding cutoff	Sigma

Allosensitization can occur following immunizing events such as pregnancy, transfusion, or transplant. In a study of sensitization to HLA antigens using subclass-specific IgG1–4 reporter antibodies in the single antigen bead assay, Lowe and colleagues showed that the subclass repertoire following unequivocal immunization to HLA antigens through pregnancy and transplantation is heterogeneous and dominated by the IgG1 subclass in 38 patients (128). IgG2 secondarily dominant following blood transfusion and failed transplant, while IgG3 was secondarily dominant following pregnancy. Generally, blood transfusion stimulated a restricted, IgG1-dominated response to HLA antigens.

The strength of pretransplant DSA is associated with ABMR and graft loss. As shown by Lefaucheur and colleagues using the traditional single antigen bead assay, the relative risk for ABMR increased significantly as the strength of preformed DSA increased (133). Patients with MFI of 3000–6000 or >6000 had greater than 60-fold or 100-fold risk, respectively, of developing AMR. Graft survival at 8 years after transplant among patients with preformed DSA >3000 MFI was only 60.6%, as compared to 78.4% for patients with preformed DSA strength of ~500–3000 MFI and 82.5% for patients negative for preformed DSA (133).

### Donor-Specific HLA Antibody Subclasses: Post-Transplant

Preliminary work, using subclass-specific IgG1–4 antibodies in flow cross-match or ELISA platforms, suggested that predominant expression of IgG1 in pretransplant sera was associated with acute rejection (134) and graft loss (132, 135). In a small study evaluating the IgG subclass of DSA using flow cytometry on donor spleen cells in kidney and liver recipients (136), one patient who lost the graft due to hyperacute rejection had high IgG3 DSA pretransplant despite a negative CDC-XM, suggesting that high titers of this subclass are potent mediators of complement-dependent rejection.

More recent data, using the subclass-specific modification of the single antigen assay, support the predominance of the IgG1 subclass in transplant patient sera (126, 127). IgG2 and IgG4 do not typically constitute a large proportion of HLA DSA (137). Several groups have attempted to further define the various subclasses as predictive biomarkers of graft pathology and outcome (127, 129, 130). Lobashevsky et al. analyzed the specificities as well as the subclasses of DSA pretransplant and post-transplant in three renal recipients using SAB modification with subclass secondaries in each patient, the proportion of different IgG subclasses were different against different antigens (138). One patient experienced rejection early post-transplant and had two DSA, both of which were about equal mixture of IgG1 and IgG2; the other two patients had good outcomes: one had IgG1, IgG2, and IgG4 DSA, whereas the other had predominantly IgG1. It is important to note that this paper highlights that each antigen can be recognized by multiple subclasses.

A cohort of post-liver transplant patients with chronic rejection and a group of patients without rejection were studied to determine if the presence of IgG subclass-specific DSA correlated with clinical state (127). The data showed that chronic rejection in liver transplant patients is correlated with the presence of DSAs of multiple subclasses, while normal graft function in the presence of DSA is correlated with DSA isolated to the IgG1 subclass. Furthermore, DSA of the IgG3 subclass was more closely associated with graft loss than DSA to other subclasses or no DSA. Everly et al. also found that the presence of IgG3 HLA DSA, particularly concurrent with IgM DSA, was predictive of allograft failure in renal transplant recipients (139).

Gao et al. first observed that IgG4 was increased in most recipients post-transplant (136). In pre- and post-transplant sera from 80 sensitized renal transplant patients, pretransplant IgG4 levels were predictive of acute ABMR in the first 30 days post-transplant, while preformed IgG4 and post-transplant day 30 IgG3 were associated with graft loss (129). In another study, sera from 125 consecutive renal transplant patients with DSA detected within the first year post-transplant evaluated for subclasses of IgG showed that IgG3 is associated with AMR, while IgG4 was associated with subclinical ABMR in protocol biopsies and late allograft injury (130). These data regarding the potential pathogenesis of IgG4 in renal transplant patients in the early post-transplant period are interesting as this subclass has been classically considered a marker of chronic antigen exposure produced due to “hyperimmunization” (140). The comment by Schaub et al. (141) is right on point – the presence of later subclasses IgG2 and IgG4 is suggestive of a more advanced humoral response with active T cell help. Donor-specific IgG4, despite its inability to activate complement, is correlated with poor graft outcome. Nevertheless, it is at this time difficult to dissect whether a predominance of IgG4 in any inflammatory disease, including transplantation, is due to the mechanism/pathogenicity of this subclass or is reflective of extensive antigen exposure and immune memory.

Given that most patients exhibit a mixture of IgG subclasses directed to HLA, several attempts have been made to evaluate whether grouping subclasses by presumed capacity to activate effector functions is able to further stratify risk. HLA DSA in sera from pre-kidney transplant patients were classified according to the presence of preformed DSA that are strong complement fixing (IgG1 and IgG3; *n* = 21/74 patients), weak/non-complement fixing (IgG2 and IgG4; *n* = 4/74 patients), or a mixture of both (containing a mixture of IgG1–4; *n* = 46/74) (126). While a trend was observed implying that patients with exclusively weak/non-complement-fixing DSA had lower incidence of ABMR at 6 months post-transplant, the incidence and histologic phenotypes of ABMR in patients displaying strong complement-fixing DSA was not significantly different from those that displayed a mixture of weak/non-complement-binding and strong complement-binding DSA. In a similar approach, Arnold et al. described the IgG subclass patterns of *de novo* DSA in adult renal transplant recipients by grouping subclasses together based on presumed complement activity. They observed that a majority of patients had exclusively “complement-fixing” IgG1 and IgG3, while the remainder had a mixture of complement-fixing and non-complement-fixing subclasses, with a very small percentage having IgG2 and IgG4 alone. ABMR was more often observed in patients with a mixture of subclasses than with IgG1/IgG3 only; however, there was no difference in graft survival between these groups (142). Interestingly, the DSA in patients with only IgG2 and IgG4 were directed exclusively against HLA class II antigens. Finally, Freitas et al. found that most patients exhibit a mixture of IgG subclass directed against donor HLA-DQ antigens, and there was no significant difference in incidence of rejection comparing patients who had IgG1 and IgG3 compared with those with a preponderance of IgG2 and IgG4 (95).

### Non-HLA Antibodies

Antibodies to non-HLA antigens on the surface of the endothelium or epithelium, aka, non-HLA antibodies, or AECA, have been identified with specificity to alloantigens, such as MICA or major histocompatibility complex class I chain-related gene B (MICB) (143, 144), or autoantigens, such as vimentin (145), cardiac myosin (CM), collagen V (ColV), agrin (146), endoglin, EGF-like repeats, Fms-like tyrosine kinase-3 ligand, ICAM-4 (69), and AT1R (67). Currently, there are only a few tests in clinical laboratories for the identification of non-HLA antibodies for transplant patients. Non-HLA antibodies to AT1R are measured by ELISA. Antibodies to MICA are measured using a MICA single antigen test (27). Donor-specific anti-endothelial cells can be measured by flow cytometry-based XM-ONE, which detects binding of IgG and IgM to peripheral blood endothelial cell precursors (147). While the isotypes and subclasses of antibodies to MICA are yet to be characterized, they do not appear to be predominantly complement fixing, at least in *in vitro* assays (148). Contradictorily, antibodies to MICA are found in both C4d^+^ and C4d^−^ ABMR (149). Seminal work by Dragun et al. (67) showed that AT1R antibodies were present in renal transplant recipients with refractory vascular rejection but with no HLA DSA [substantiated by Reinsmoen et al. (150)], and the same group went on to show that these antibodies were predominantly IgG1 and IgG3 subclasses (151). Interestingly, however, histological manifestations of AT1R-mediated graft dysfunction do not typically include positive C4d staining, suggesting that injury *via* anti-AT1R antibodies might be complement independent, despite a predominance of strongly complement-fixing subclasses. As mentioned above, bivalent antibodies may act agonistically; and clinically AT1R-mediated graft dysfunction can present with hypertension and histologically with MVI (152). Using XM-ONE, patients with positive endothelial progenitor cross-match experienced increased rejection (majority were C4D^−^ and classified as cellular rejection) and higher serum creatinine (153). AECA that bind ECP were found to be present in about 60% of patients tested (HLA DSA^−^) and were primarily of the IgG2 and IgG4 subtypes (69, 131, 154).

### Limitations

Several caveats to the modification of single antigen testing to detect IgG subclasses warrant discussion. First, the IgG 1 and 2 subclass-specific antibodies exhibit non-specific binding to single antigen beads coated with the alternate antigen. For example, the IgG1 subclass-specific antibody cross-reacts with the IgG2 antigen at an MFI that is about 4.42% of the value observed when it specifically binds its target on an IgG1 coated bead (128). Cross-reactivity is observed between the IgG2 subclass-specific antibody and the IgG1 antigen ranging from 3 to 15%; however, the IgG3 and 4 antibodies appear to be less cross-reactive (126, 128). Second, the IgG1–4 antibodies bind single antigen beads coated with their cognate antigen with different strengths (IgG1 > IgG2 > IgG3 > IgG4), suggesting that the antibodies have different affinity for their target antigens (128). Third, the concentration of the different IgG subclasses cannot be directly compared to infer relative abundance of each subclass. Fourth, the sensitivities of the traditional single antigen assay and modified subclass-specific assay are different. Notable is that a minor proportion of antibodies detected using total IgG were not detected with any four subclass reagents. Antibodies <2000 MFI in the traditional single antigen bead assay can be negative in the subclass-specific assay (126, 129). Finally, a review of the literature shows that the methods for defining the threshold for positivity are vastly different (Table 2). While not diminishing the findings, these limitations, as well as the inherent semi-quantitative nature of the Luminex assay, do restrict the analyses to the presence or absence of IgG1–4 against a specific HLA antigen and currently do not reflect the titer or concentration of any subclass.

Another notable consideration is the variability of induction therapy among studies of IgG subclass distribution of HLA DSA, including basiliximab (129, 130), ATG (130), OKT3, thymoglobulin, or daclizumab (155). The impact of different immunosuppression and induction therapies on isotype switching has not been thoroughly evaluated. What little is known is discussed below and suggests that both maintenance immunosuppression and induction treatments influence B cell class switching.

## B Cell Differentiation and Class Switching

### IgG Subclasses in the Context of Protective Immunity

Exposure to different types of antigens stimulates dramatic skewing of IgG subclasses. Protein antigen leads to T-cell-dependent isotype switching to IgG1 and IgG3 that tend to dominate responses to viral and bacterial protein antigens. The importance of the diversity of Ig isotypes and subclasses is apparent from phenotypes of humans with selective subclass deficiencies, monoclonal gammopathy, and multiple myeloma. IgG3 or IgG1 deficiency increases susceptibility to bacterial respiratory tract infections. By the classical paradigm, bacterial and yeast polysaccharide antigens stimulate T-independent IgM responses and IgG2 production. Other evidence suggests that antibody responses to glycolipids and glycoproteins may obtain some help from the T cell compartment. IgG2 is predominantly produced in response to pneumococcal polysaccharides and encapsulated antigens (156, 157). The most infectious complications are seen in individuals with IgG2 deficiency, who demonstrate heightened susceptibility to respiratory bacterial infections, due to impaired responses to polysaccharides of encapsulated bacteria. IgG4 is often produced in settings of non-infectious immunity, such as allergy; immune therapy in beekeepers and allergic individuals promoted switching to IgG4 and relieved symptoms of allergy (158, 159). IgG4 is also produced in response to helminth and filariasis parasitic infections (which, like allergy, also elicit IgE) (160, 161). The clinical significance of deficiency of IgG4 is unclear.

### Mechanisms of Class Switching

Immunoglobulins are tetrameric proteins composed of two heavy chains and two light chains connected by disulfide bonds. The IgG subclasses are numbered in order of abundance in circulation rather than order on the genome; in the human germline, the genes encoding the constant regions are ordered μ, δ, γ3, γ1, α1, γ2, γ4, ε, and α2. With the exception of membrane IgM and IgD in mature naïve B cells, a single B cell can only express one isotype of immunoglobulin at a time.

**Figure 4:**
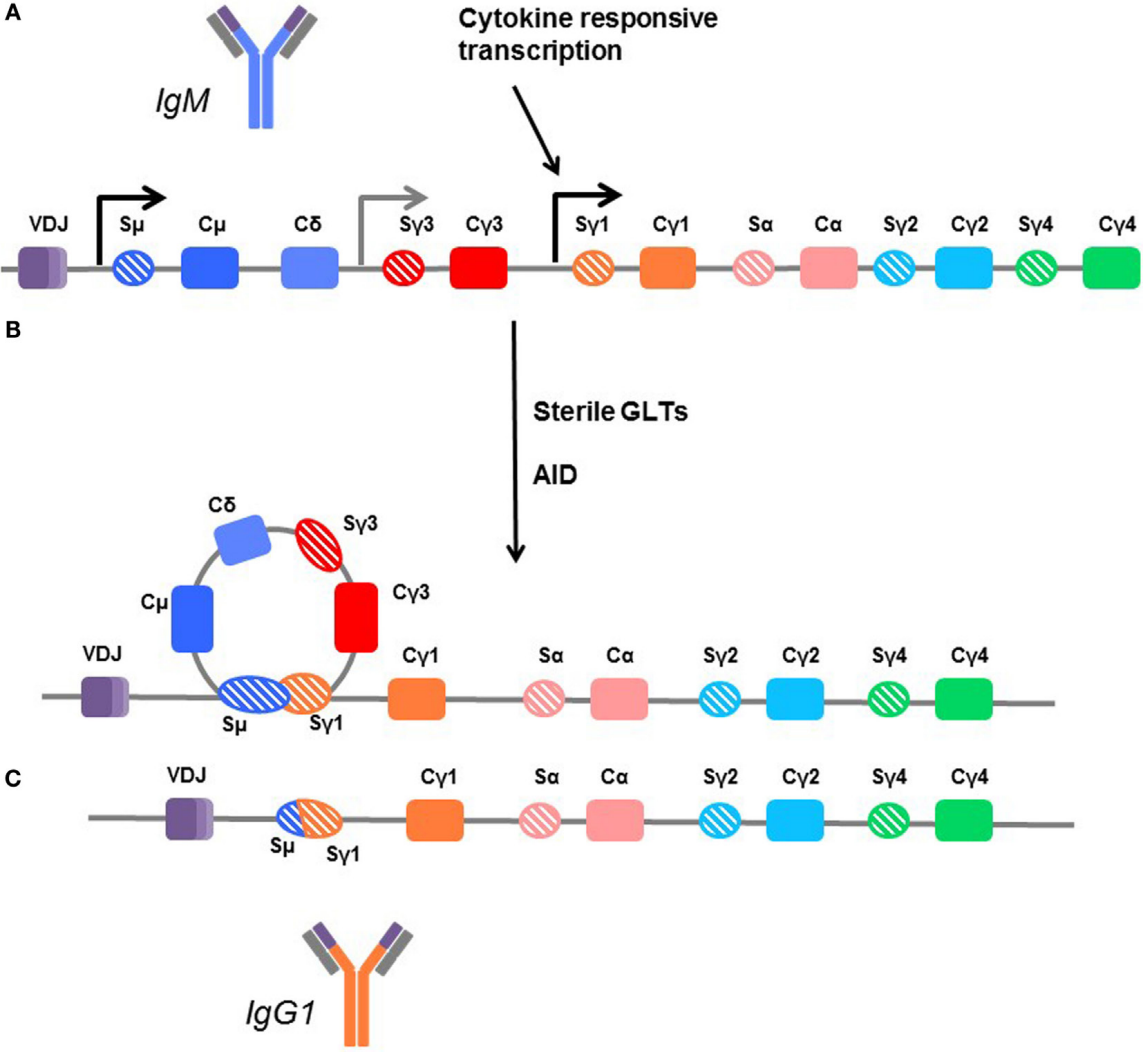
**Mechanisms of class switching**. **(A)** In unswitched human B cells, the germline arrangement of heavy chain immunoglobulin genes is ordered by the variable region (VDJ) followed by the constant regions for IgM (Cμ), IgD (Cδ), IgG3 (Cγ3), IgG1 (Cγ1), IgA1 (Cα), IgG2 (Cγ2), and IgG4 (γ4). Each is flanked by a switch region (e.g., Sγ3). These regions are sites of transcription initiation and produce sterile germline transcripts (GLTs). **(B)** Transcription from the flanking switch regions is thought to make the DNA accessible to enzymes, such as activation-induced cytidine deaminase (AID), which facilitates recombination between switch regions, looping out the interior constant region genes. **(C)** In this example, the B cell is switching directly from IgM (dark blue) to IgG1, and the Cμ, Cδ, and Cγ3 genes are removed so that the variable region can be directly fused with the Cγ1 region to form the IgG1 molecule (orange). Thus, B cells which are isotype switched can only further isotype switch the remaining subclasses downstream. Cγ4 is terminal.

The genes encoding the various isotypes and subclasses are flanked by switch sites, each with its own promoter. The promoters for the switch regions contain binding sites for cytokine-responsive transcription factors, bridging exogenous signaling with isotype selection. Many cytokines, mostly Th2-associated, have been implicated in isotype and subclass specification, including IL-4, IL-13, IL-21, and IL-6 (162–165). However, no dedicated and unique switch factor has yet been identified for any given immunoglobulin heavy chain. Transcription of these switch regions produces a sterile germline RNA transcript (GLT). Subsequently, the variable region is joined with a downstream CH segment encoding a different Ig isotype to generate a new heavy chain (Figure 4).

**Figure 5:**
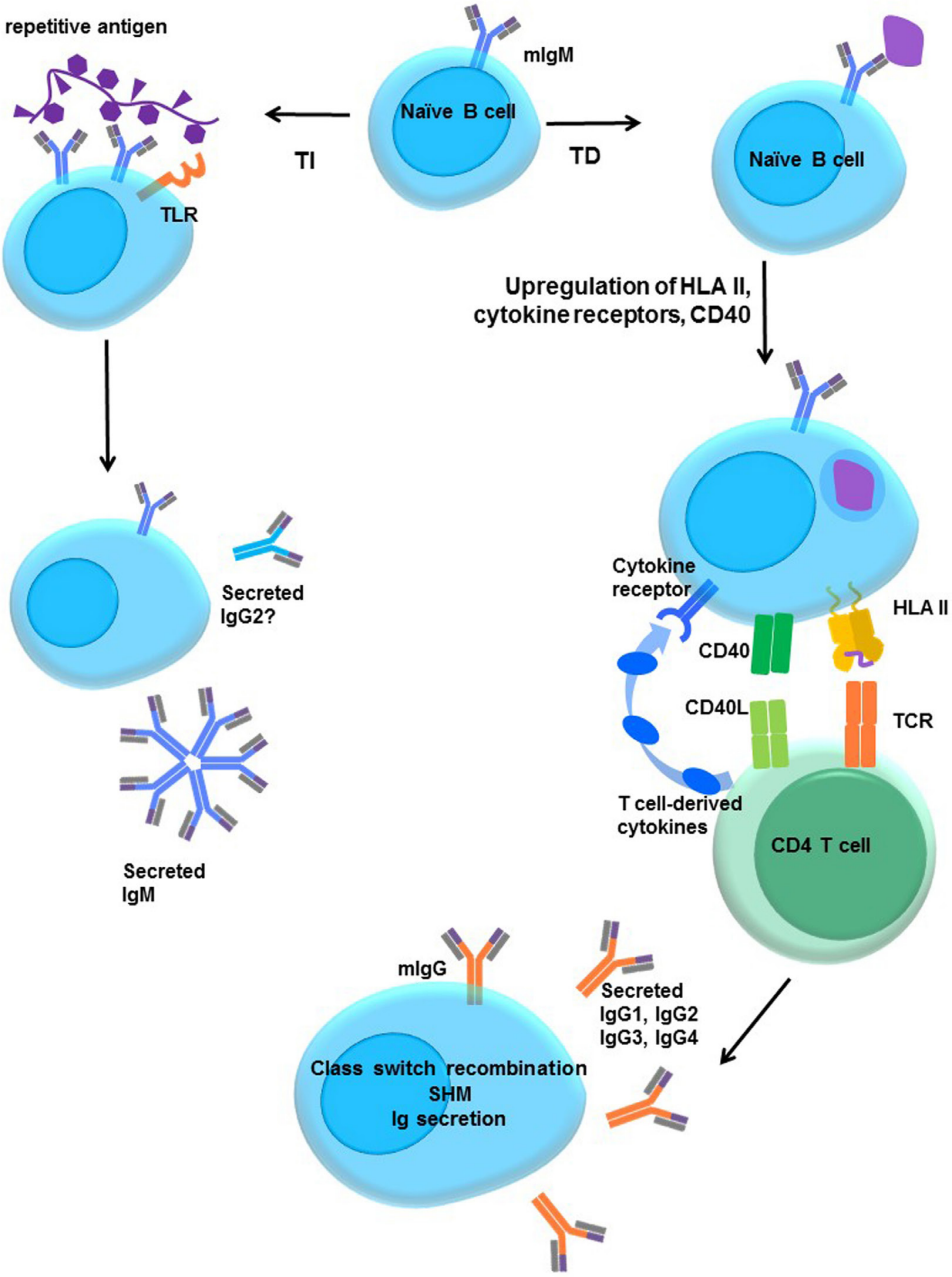
**Activation of naïve B cells**. Upon encountering cognate antigen through the membrane-associated IgM BCR, engagement of the BCR triggers intracellular signaling. On its own, moderate BCR cross-linking is not sufficient to induce proliferation, but cells do upregulate costimulatory molecules, such as CD40 and B7 (CD80/CD86), and increase antigen processing and presentation. The B cell may internalize the antigen into endosomal vesicles (and downregulates surface IgM), enabling it to process and present the antigen in HLA. B cells upregulate HLA II, cytokine receptors, costimulatory molecules CD40, B7-1, and B7-2, enter the cell cycle, and increase expression of prosurvival genes, and protein translation machinery. T-dependent protein antigens can be internalized and processed into peptides for presentation to CD4 T helper cells in HLA molecules. CD4 T cells provide additional costimulation *via* CD40L and expression of cytokines, such as IL-21, IL-10, IL-13, or IL-4. Integration of the BCR, CD40, and cytokine signaling stimulates class switch recombination, and the B cell ultimately switches from production of only membrane IgM and IgD to secretion of other isotypes such as IgG. T-independent antigens, such as those with repetitive motifs, extensively cross-link the BCR but are difficult to internalize. Glycolipid or polysaccharide antigens cannot be presented in classical HLA molecules for CD4^+^ T cell help. Concurrent signals may derive from Toll-like receptor (TLR) stimulation by antigens, NKT or αβ T cell help, leading to enhanced B cell activation and secretion of IgM or IgG2.

Mature naïve B cells express membrane IgM and IgD. B cell activation is step-wise, and a simple schematic is shown in Figure 5. Formation of a synapse between T and B cells facilitates CD40–CD40L (CD154) interactions that prime the B cell. Cytokines signal the B cell to switch the isotype and subclass of immunoglobulin, and to secrete Ig. To isotype switch, the B cell must rearrange its DNA, linking the functional variable (VDJ) region of the heavy chain to one constant region heavy chain gene in a process called class switch recombination (CSR).

The first isotype of immunoglobulin that is produced is membrane-associated IgM (mIgM). IgM is also the first to be secreted upon B cell activation, as the switch from mIgM to secreted IgM requires only a change in mRNA splicing of the μ transcript to exclude the CH4 transmembrane domain. Upon primary exposure to an antigen, B cells will secrete IgM within 4–5 days, peaking by about 1–2 weeks. Expression of IgG, IgA, and IgE, however, usually require further, division-linked, and genomic DNA rearrangement and do not appear until about a week after initial exposure. With a few exceptions, CSR is CD4 T cell dependent.

Seminal work by Lechler and others demonstrated that immune recognition of allogeneic proteins occurs through three major pathways: direct, indirect, and semidirect allorecognition. The details of these pathways have been excellently reviewed elsewhere (166, 167). The direct pathway of allorecognition represents and important, although apparently transient (168, 169), mechanism of T cell response to solid organ allografts. Current paradigm holds that CD4 T cells recognize MHC class I alloantigens *via* the indirect pathway and are indispensable for alloantibody-mediated rejection (170–174). Shed alloantigens, including soluble MHC, may be taken up by host antigen-presenting cells, processed, and allopeptides presented in the context of host MHC II. Whole soluble donor MHC stimulates a more robust alloantibody response than immunization with MHC peptides. B cells themselves are involved in the indirect presentation of donor antigen and activation of CD4 T cells (175, 176).

Given what is known about antibody isotype specification, how might alloreactive B cells be driven to form anti-HLA antibodies of a given IgG subclass? The cytokine milieu and availability of costimulation are critical for B cell antibody generation and the environment under which class switching occurs during transplantation has yet to be explored experimentally. As mentioned above, it is conceivable that solid organ transplantation represents an extreme form of chronic antigen stimulation that ultimately results in the formation of IgG4 alloantibodies. One important feature of B cell activation unique to the post-transplant setting is that it occurs under the veil of maintenance immunosuppression. A few experimental studies have attempted to address the impact of immunosuppressive drugs on the mechanisms of class switching.

### Effects of Immunosuppression

The frequency of DSA in patients with medication non-adherence is much higher than in those without – ~70% at 12 years (1, 2, 177) – suggesting that current immunosuppressive regimens impact humoral allosensitization.

In rodents, mycophenolate mofetil (MMF) treatment significantly altered IgG subclass distribution and reduced autoantibody production and development of systemic autoimmunity (178, 179). In an *in vitro* study with human T and B cells, cyclosporine, mycophenolic acid (MPA), rapamycin, and, to a lesser extent, tacrolimus inhibited T cell proliferation; however, activation of T cells, as measured by CD25 and CD69, was unaffected. All of these drugs slightly dampened CD154 (CD40L) expression but significantly reduced Tfh cell differentiation and suppressed cytokines implicated in B cell help (180, 181). However, if T cells were first activated and then subsequently exposed to immunosuppression, only rapamycin and MPA prevented IgM production by B cells. Therefore, memory T cells may still be capable of stimulating B cell responses even under suppression by tacrolimus (180).

In mice, costimulation of T cells through B7-1 (CD80) and B7-2 (CD86) are critical for antibody responses and particularly for IgG isotype switching *via* a non-redundant role with CD40 (182). Belatacept is a CTLA-4 fusion protein that blocks T cell costimulation *via* B7. In a non-human primate transplant model, costimulatory blockade with CTLA-4 fusion proteins reduces *de novo* alloantibody production in mice and non-human primate models (183–185). In human renal transplant recipients, the BENEFIT trial revealed that lowly HLA-sensitized patients treated with Belatacept had lower reduced frequency of *de novo* donor-specific antibody production compared with the control cyclosporine arm (186, 187). The B cell compartment in Belatacept-treated recipients was skewed toward naïve and transitional phenotypes (188). It is notable that patients in these trials were either DSA-free or low risk for preformed HLA DSA (186, 187). The impact of Belatacept on B cell activation and isotype switching in allosensitized patients might be less efficacious due to reduced costimulation requirements of memory immune cells [discussed in Ref. (189)].

There is also evidence that immunosuppressive drugs might alter isotype switching *via* direct effects on B cells. Prior depletion of B cells using rituximab reduced IgM and IgG1 responses after vaccination early after treatment, with a durable inhibition of IgM seen 6–10 months after vaccination (190). mTOR is central to the ability of B cells to proliferate and regulates CSR, and rapamycin has an impact on IgG production and plasmablast differentiation (191). Leflunomide is an immunosuppressant often used off-label in transplant recipients with viremia, such as with CMV or BK virus. Leflunomide acts on both T and B cells through inhibition of the JAK/STAT pathway critical for B cell signaling. Leflunomide inhibits IgG production in rodents, including reduction of donor-specific antibodies in transplant models (192, 193).

In summary, multiple immunosuppressive agents have been demonstrated to impact isotype switching by B cells in *in vitro* and animal models. However, definitive evidence from systematic trials in humans is lacking to demonstrate differential effects on donor-specific IgG subclass production.

## Closing Remarks

Clinical experience confirms that all donor-specific antibodies are *not* created equal. ABMR is a wide spectrum of graft injury from complement-mediated hyperacute rejection, to histological injury without graft dysfunction, to fibrotic chronic rejection and vasculopathy. Whether further discrimination of pathogenic DSA can be provided by complement fixation *in vitro* or by identifying the subclass(es) present remains to be determined. A majority of individuals pre- and post-transplant exhibit antibodies against HLA that are a mixture of IgG subclasses. Cumulatively, studies to date indicate that donor-specific IgG3 may be most relevant to acute antibody-mediated injury, while IgG4 DSA might signify alloimmune memory and correlate with subclinical and chronic rejection. However, IgG1 is also found in nearly all cases, indicating that heterogeneous subclass responses are the norm. These studies highlight the complexity of the alloimmune response and underscore the constraints on interpreting the relevance of DSA subclass repertoire in graft outcome, since exclusive skewing of donor-specific antibodies to one single subclass is rarely observed.

Three challenges must be overcome in order to identify characteristics of pathogenic DSA. First, laboratories are faced with the task of developing reliable, informative, and cost-effective assays that can detect differences in effector functions or other features of HLA antibodies. Second, the mechanisms of graft injury by different subclasses of HLA antibodies should be confirmed in experimental transplant models and *in situ* in allografts. To date, we can only postulate that in the setting of transplantation, anti-HLA IgG3 and IgG1 might elicit extensive complement activation and ADCC, while IgG2 and/or IgG4 may induce only HLA signaling in the allograft with little complement activation or FcγR-mediated functions. Much of the knowledge of antibody effector functions is derived from infectious disease and autoimmune and cancer research, but little work has evaluated the capacity of different human HLA IgG subclasses to elicit inflammation and injury during ABMR. Finally, few interventions exist for the treatment of ABMR, and their impact on class switching of alloreactive B cells is mostly uncharacterized. Therapies might be designed to manipulate the humoral alloimmune response to produce one subclass rather than another, but more effort is needed to understand the details of isotype specification by cytokines and other signals.

## Author Contributions

NV and MH contributed to the outline and writing of the manuscript. ER contributed to the outline and critical review of the manuscript.

## Conflict of Interest Statement

The authors declare that the research was conducted in the absence of any commercial or financial relationships that could be construed as a potential conflict of interest.
